# Synthesis of polyfunctionalized dihydro-2-oxypyrroles catalyzed by 1,2,3,5-tetrakis(carbazol-9-yl)-4,6-dicyanobenzene (4CzIPN) as a novel donor-acceptor fluorophore

**DOI:** 10.1038/s41598-022-20689-4

**Published:** 2022-10-07

**Authors:** Farzaneh Mohamadpour

**Affiliations:** School of Engineering, Apadana Institute of Higher Education, Shiraz, Iran

**Keywords:** Chemistry, Photocatalysis

## Abstract

We developed a green radical synthesis method for polyfunctionalized dihydro-2-oxypyrroles based on the Michael–Mannich cyclocondensation of amines, dialkyl acetylenedicarboxylates, and formaldehyde. To generate a renewable energy sources from visible light, a PCET (proton-coupled electron transfer) photocatalyst was used in an ethanol solution in an air environment and at room temperature. In this study, we aim to develop an inexpensive and easily accessible novel donor–acceptor (D–A) fluorophore. Besides its speed-saving features and ease of use, the carbazole-based photocatalyst (4CzIPN) also shows high yields, energy-efficient, and is environmentally friendly. In this way, it is possible to monitor changes in chemical and environmental variables over time. The variety of yields is pretty uniform (84–97%, average 92.3%), and the kind of response times be very speedy (15–25 min, average 17.6 min), and the element noted within the dialogue is that the system tolerates a variety of donating and withdrawing functional groups, at the same time as nevertheless giving very fast rate and tremendous yields. A study of polyfunctionalized dihydro-2-oxypyrroles was conducted to calculate the turnover number (TON) and turnover frequency (TOF). Gram-scale cyclization proves that it can be applied to industry in a practical manner.

## Introduction

Both academia and industry are interested in developing clean, economical, and efficient chemical processes from a green and sustainable chemistry perspective. By forming versatile open shell reactive species, radical chemistry has emerged as a powerful tool for rapidly constructing complicated organic molecules. Thermolysis, radiation, photolysis, electrolysis, and redox systems can all be used to trigger radical reactions. A particularly clean and promising method among these strategies is photocatalysis, which has been extensively applied to radical chemistry^1–10^.

A parallel to these discoveries is the renaissance of visible light photo-redox catalysis in organic synthesis, providing novel opportunities for the design of synthetic routes through efficient light-mediated transformations. These reasons have led to the widespread use of photo-redox catalysis in the synthesis of building blocks, pharmaceuticals, and the total synthesis of complex natural products^11^. The electron-transfer process is enabled in a significant percentage of these transformations by the use of ruthenium (II) and iridium (III) complexes as photocatalysts^12–14^.

**Figure 1:**
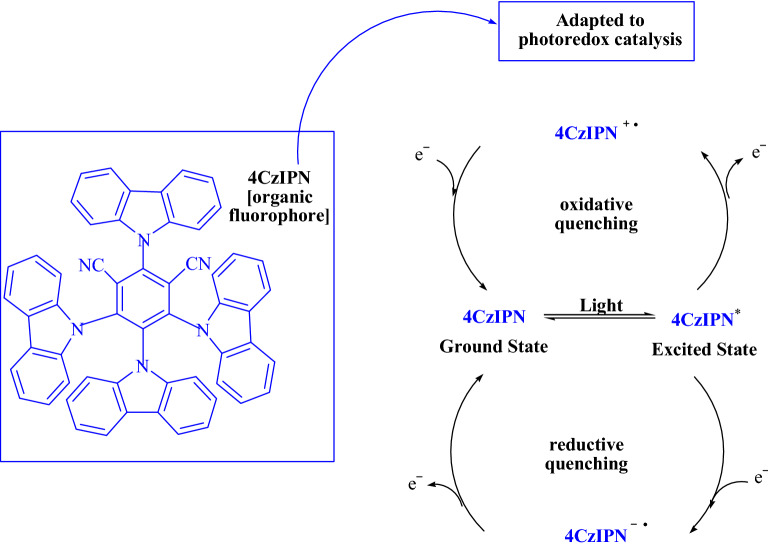
The 4CzIPN is capable of performing photocatalytic cycles^11^.

According to Zeitler et al.^15^, libraries of derivatives of 4CzIPN-type photocatalysts possess diverse electrochemical properties and may prove beneficial for developing unique reactions in the future^14^. 1,2,3,5-Tetrakis(carbazol-9-yl)-4,6-dicyanobenzene (4CzIPN), which combines carbazolyl as a donor and dicyanobenzene as an electron acceptor, is a novel donor–acceptor (D–A) fluorophore (Fig. 1 shows its photocatalysis cycles^11^). The excellent redox window, environmental and economic sustainability, as well as their broad applicability and established electronic properties, make 4CzIPN an attractive metal-free photocatalyst^6,14,16^.

For environmentally friendly organic chemical synthesis, visible light irradiation is a reliable technology due to its large energy reserves, cheap cost, and renewable energy sources^17–19^.

**Figure 2:**
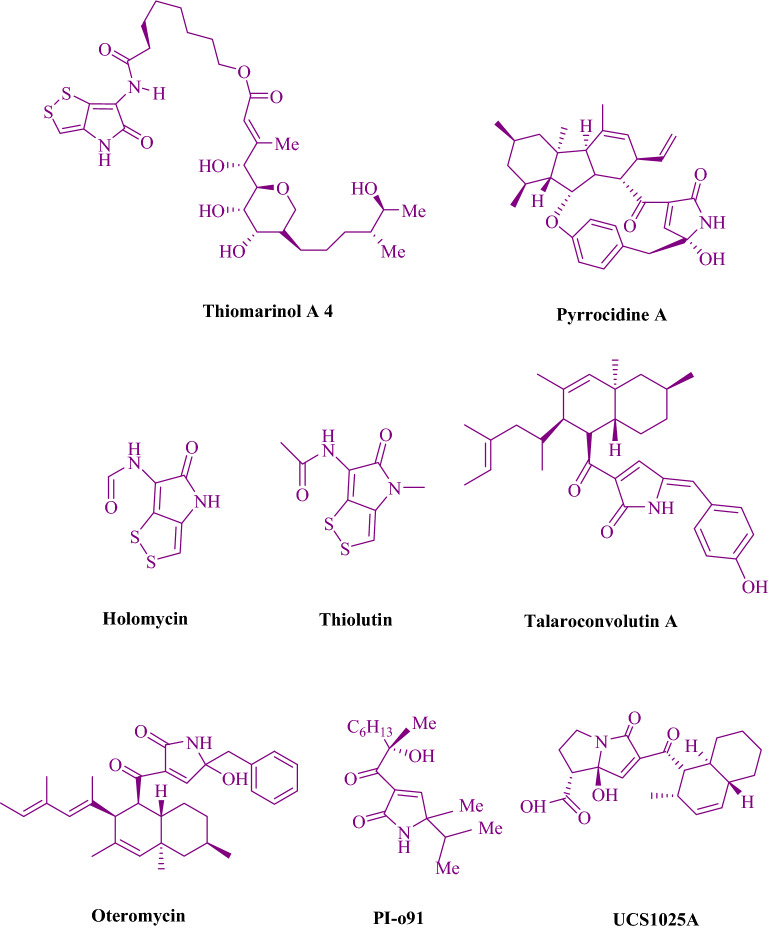
Pharmaceutically active oxypyrrole rings.

Oxypyrrole rings are regarded as being bio- and pharmacologically interesting (Fig. 2). The human cytomegalovirus protease (HCMV) is one example with pyrrole rings^20^, in addition, human cytosolic carbonic anhydrase isozymes^21^, PI-091^22^, Oteromycin^23^, cardiac cAMPphosphodiestrase^24^, and most alkaloids^25^.

There are several synthetic techniques that can be used to make polyfunctionalized dihydro-2-oxypyrroles such as methylene blue^26^, I_2_^27^, glycine^28^, AcOH^29^, Cu(OAc)_2_·H_2_O^30^, Fe_3_O_4_@nano-cellulose–OPO_3_H^31^, tartaric acid^32^, nano-Fe_3_O_4_@SiO_2_/SnCl_4_^33^, glutamic acid^34^, graphene oxide^35^, caffeine^36^, 2,6-pyridinedicarboxylic acid^37^, saccharin^38^, BiFeO_3_ nanoparticles^39^, and CoFe_2_O_4_@SiO_2_@IRMOF-3^40^. Consequently, there is a shortage of metal catalysts, high reagent costs, difficult reactions, and poor yields, which increases reaction duration and impacts waste management. As well, homogeneous catalysts are challenging to isolate from reaction mixtures. In a green medium, we have recently created heterocyclic compounds using photocatalysts. Fluorophore organic dye photo-redox catalysts are also accessible and affordable, according to the study. As a result of this methodology, donor–acceptor (D–A) has emerged as a potent organo-photocatalyst. In our research, we were particularly focused on 1,2,3,5-tetrakis(carbazol-9-yl)-4,6-dicyanobenzene (4CzIPN) due to its photophysical and photochemical properties. In response to this situation, carbazolyl dicyanobenzenes (CDCBs) have emerged as a novel donor–acceptor (D–A) fluorophore with intriguing photoelectric performance, expanding organic chemists’ toolbox of photocatalysts. In an organic dye compound containing carbazole as donor and dicyanobenzene as acceptor, a significant redox window was observed, as well as excellent chemical stability and a wide range of applications.

4CzIPN is a novel carbazole-based photocatalyst that has already been characterized as a proton-coupled electron transfer (PCET) photocatalyst. This process uses Michael–Mannich cyclocondensation, which uses amines, dialkyl acetylenedicarboxylates, and formaldehyde, which can also utilize visible light as a renewable energy source and an air atmosphere in an ethanol solution at room temperature. In spite of the fact that it was completed within budget, on schedule, and without a hitch.

A well-known Michael–Mannich cyclocondensation reaction for the synthesis of polyfunctionalized dihydro-2-oxypyrroles become developed in an eco-safe manner with the 4CzIPN is a novel carbazole-based photocatalyst. In keeping with the consequences, it turned into discovered that this technique is a fruitful one-pot technique underneath quite powerful and facile response situations. Exceedingly fast conversion with extremely good yield using renewable energy supply makes this protocol appealing to inexperienced chemists. Multigram scale as much as 50 mmol and synthesis of real-global drug-API denotes its pharmaceutical importance. Similarly, key capabilities consist of a clean experimental setup, huge substrate tolerance, budget-friendly, easy art work-up techniques in the absence of tedious separation strategies, and minimized amount of waste for every organic transformation. The kind of yields is quite uniform (84–97%, average 92.3%), and the form of response times could be very rapid (15–25 min, average 17.6 min), and the point cited in the discussion is that the technique tolerates pretty a number donating and withdrawing functional groups, even as however giving awesome yields. The response is fairly insensitive to the person of the substituents.

## Experimental

### General

A 9100 electrothermal device was used to determine the melting points of all compounds. Furthermore, CDCl_3_ was used to record ^1^HNMR, and ^13^CNMR with Bruker DRX-400, DRX-300, and DRX-100 Avance tools. Mass spectra were obtained with an Agilent Innovation (HP) spectrometer operating at 70 eV. There were large quantities of these reagents given by Fluka, Merck, and Acros, and they were quickly used.

### A step-by-step procedure for producing polyfunctionalized dihydro-2-oxypyrroles (5a–u)

Amine **1** (1.0 mmol) and dialkyl acetylenedicarboxylate **2** (1.0 mmol) in the presence of 4CzIPN (1 mol%) was stirred for 15 min at room temperature in EtOH (3 mL). While adding formaldehyde **4** (1.5 mmol) and amine **3** (1.0 mmol), the reaction mixture was stirred at room temperature. Recording the responses was done using thin layer chromatography (TLC). TLC was carried out with silica gel as the stationary phase utilizing EtOAc/*n*-hexane (1:2) as an eluent. This pure chemical was then purified without additional purification by screening and washing with ethanol after the reaction. In terms of pharmaceutical process R&D, we are interested in determining if we can produce the aforementioned substances on a gram scale. The following four substances were used in one experiment: 50 mmol: 6.37 g 4-chloroaniline, 37.5: 1.12 g mmol formaldehyde, and 25 mmol: 3.55 g dimethyl acetylenedicarboxylate (DMAD). The product was collected using a conventional filtration method after 20 min of reaction time. On the basis of the ^1^HNMR spectrum, it appears that the compound is spectroscopically pure. We classified the products based on their spectroscopic data (^1^HNMR, ^13^CNMR, and mass). Detailed information is available in the “Supporting Information” file.

## Results and discussion

**Figure 3:**
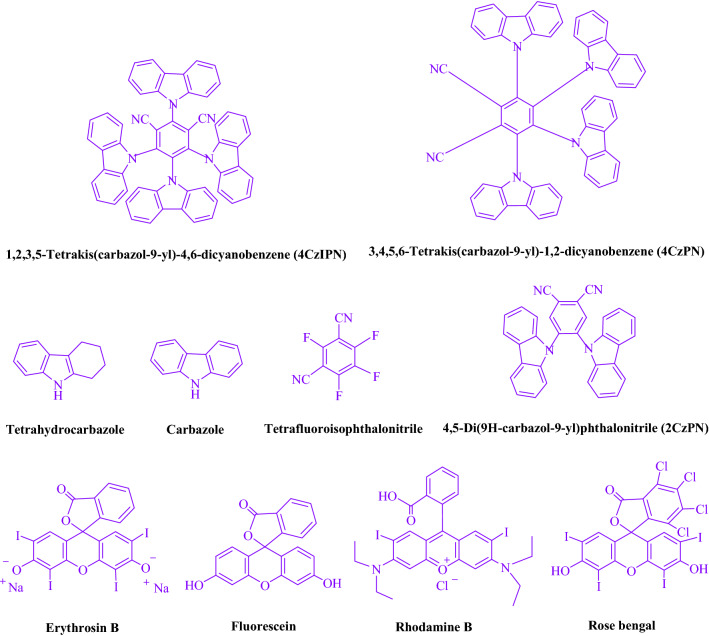
Catalyst performance in this investigation was examined.

**Figure 4:**
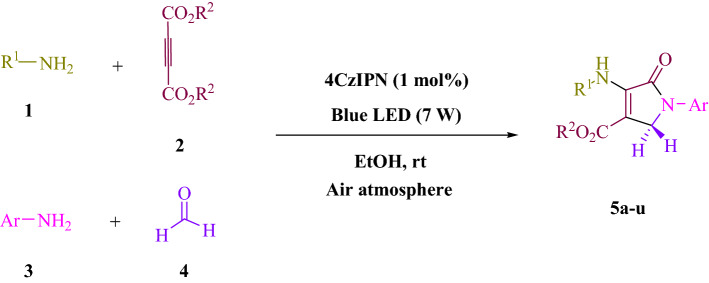
An approach to creating polyfunctionalized dihydro-2-oxypyrroles.

The purpose of this experiment was to examine the reaction between formaldehyde (1.5 mmol), aniline (2 mmol), and dimethyl acetylenedicarboxylate (DMAD) (1 mmol) in EtOH (3 mL). A trace amount of **5a** at rt was produced by 3 mL of EtOH for 60 min without photocatalyst (Table 1, entry 1). The reaction was intensified by adding numerous additional photocatalysts. As shown in Fig. 3, these compounds included 4CzIPN, 4CzPN, tetrahydrocarbazole, carbazole, tetrafluoroisophthalonitrile, 2CzPN, erythrosin B, fluorescein, rhodamine B, and rose bengal. A yield of 19–97% can be obtained by using this process for the synthesis of **5a** (Table 1). By achieving these results, 4CzIPN was able to perform better. In Table 1, entry 3, it is shown that 97% of the yield was obtained by using 1 mol% 4CzIPN. Table 2 shows that DCM, solvent-free, DMSO, DMF, THF, and H_2_O yielded significantly lower values. As a result EtOAc, CH_3_CN, and MeOH yielded more and accelerated the reaction. The rate and yield of the reaction in EtOH were both high. Using the data from Table 2’s entry 6, 97% yield was achieved. Studies have used various light sources to investigate the impact of blue light on yield (Table 2). As a result of controlling the test without the light source, **5a** was detected in the trace. As a result of the study, 4CzIPN and visible light are necessary for the production of product **5a**. Based on 3 W, 7 W, and 10 W blue LED intensity levels, the optimal settings were determined. Based on the results obtained with blue LEDs (7 W), the best results were obtained (Table 2, entry 6). Several substrates were subjected to tests under ideal conditions (Table 3 and Fig. 4). Aniline substitution had no influence on the reaction outcome (Table 3). There were no restrictions on halide substitutions in this reaction. The current state of the reaction allows both reactions of electron-donating functional groups as well as reactions of electron-withdrawing functional groups. There is a great deal of yield potential for aliphatic and benzylic amines. There is a similar reactivity between dimethyl acetylenedicarboxylate (DMAD) and diethyl acetylenedicarboxylate (DEAD).

**Table 1:** We provide an optimization table for photocatalysts used in **5a** production^a^.


Entry	Photocatalyst	Solvent (3 mL)	Time (min)	Isolated Yields (%)
1	–	EtOH	60	Trace
2	4CzIPN (0.5 mol%)	EtOH	15	81
3	**4CzIPN (1 mol%)**	**EtOH**	**15**	**97**
4	4CzIPN (1.5 mol%)	EtOH	15	97
5	4CzPN (1 mol%)	EtOH	15	52
6	Tetrahydrocarbazole (1 mol%)	EtOH	15	23
7	Carbazole (1 mol%)	EtOH	15	34
8	Tetrafluoroisophthalonitrile (1 mol%)	EtOH	15	19
9	2CzPN (1 mol%)	EtOH	15	41
10	Erythrosin B (1 mol%)	EtOH	25	54
11	Fluorescein (1 mol%)	EtOH	25	59
12	Rhodamine B (1 mol%)	EtOH	25	46
13	Rose bengal (1 mol%)	EtOH	25	63

Significant values are in [bold].

^a^Reaction conditions: a blue LED (7 W) is used along with a number of photocatalysts and formaldehyde (1.5 mmol), aniline (2 mmol), and dimethyl acetylenedicarboxylate (DMAD) (1 mmol) at rt.

**Table 2:** There is a table that shows the optimal concentrations of solvent and visible light for the synthesis of **5a**^a^.


Entry	Light source	Solvent (3 mL)	Time (min)	Isolated yields (%)
1	Blue light (7 W)	DCM	40	22
2	Blue light (7 W)	EtOAc	15	70
3	Blue light (7 W)	CH_3_CN	15	75
4	Blue light (7 W)	–	35	45
5	Blue light (3 W)	EtOH	15	88
6	**Blue light (7 W)**	**EtOH**	**15**	**97**
7	Blue light (10 W)	EtOH	15	97
8	Green light (7 W)	EtOH	15	90
9	–	EtOH	60	Trace
10	White light (7 W)	EtOH	15	86
11	Blue light (7 W)	MeOH	15	68
12	Blue light (7 W)	DMSO	35	32
13	Blue light (7 W)	DMF	40	29
14	Blue light (7 W)	THF	40	27
15	Blue light (7 W)	H_2_O	35	38

Significant values are in [bold].

^a^Reaction conditions: at rt, 4CzIPN (1 mol%) was mixed with formaldehyde (1.5 mmol), aniline (2 mmol), and dimethyl acetylenedicarboxylate (DMAD) (1 mmol).

**Table 3:** As a novel donor–acceptor (D–A) fluorophore, carbazole-based photocatalyst (4CzIPN) is used to create polyfunctionalized dihydro-2-oxypyrroles.


5a (15 min, 97%) Mp. 154–156 °C Lit. 155–156 °C^27^	5b (15 min, 95%) Mp. 140–142 °C Lit. 138–140 °C^29^
5c (15 min, 95%) Mp. 171–173 °C Lit. 174–176 °C^37^	5d (15 min, 94%) Mp. 151–153 °C Lit. 153–154 °C^31^
5e (20 min, 90%) Mp. 172–173 °C Lit. 170–172 °C^36^	5f (20 min, 87%) Mp. 165–167 °C Lit. 166–168 °C^36^
5g (15 min, 94%) Mp. 58–60 °C Lit. 60 °C^27^	5h (20 min, 91%) Mp. 96–98 °C Lit. 95–97 °C^37^
5i (20, 84%) Mp. 180–182 °C Lit. 179–181 °C^37^	5j (25 min, 85%) Mp. 167–169 °C Lit. 169–171 °C^29^
5k (20, 93%) Mp. 139–141 °C Lit. 140–141 °C^29^	5l (20 min, 91%) Mp. 132–134 °C Lit. 130–132 °C^29^
5m (15 min, 92%) Mp. 122–124 °C Lit. 124–125 °C^31^	5n (15, 94%) Mp. 101–103 °C Lit. 102–104 °C^31^
5o (20, 88%) Mp. 118–120 °C Lit. 120–121 °C^27^	5p (20, 91%) Mp. 100–102 °C Lit. 99–101 °C^28^
5q (15 min, 95%) Mp. 159–161 °C Lit. 161–163 °C^34^	5r (15 min, 96%) Mp. 172–174 °C Lit. 171–172 °C^34^
5s (15 min, 97%) Mp. 179–181 °C Lit. 178–180 °C^28^	5t (15 min, 94%) Mp. 132–134 °C Lit. 131–132 °C^29^
5u (20, 96%) Mp. 169–171 °C Lit. 168–170 °C^34^

A turnover frequency (TOF) and turnover number (TON) are provided in Table 4. There are two types of yield: TON = Yield/Amount of catalyst (mol) and TOF = Yield/Time/Amount of catalyst (mol). Catalyst efficiency increases with higher TON and TOF values due to less catalyst required to boost yields.

**Table 4:** In order to calculate the turnover number (TON) and turnover frequency (TOF), we made the following calculations.

Entry	Product	TON	TOF	Entry	Product	TON	TOF
1	**5a**	97	6.4	12	**5l**	91	4.5
2	**5b**	95	6.3	13	**5m**	92	6.1
3	**5c**	95	6.3	14	**5n**	94	6.2
4	**5d**	94	6.2	15	**5o**	88	4.4
5	**5e**	90	4.5	16	**5p**	91	4.5
6	**5f**	87	4.3	17	**5q**	95	6.3
7	**5g**	94	6.2	18	**5r**	96	6.4
8	**5h**	91	4.5	19	**5s**	97	6.4
9	**5i**	84	4.2	20	**5t**	94	6.2
10	**5j**	85	3.4	21	**5u**	96	4.8
11	**5k**	93	4.6				

There is a high TON = 97 and TOF = 6.46 for catalyst **5a**, and a high TON = 95 and TOF = 6.33 for catalyst **5b**, which is compared to the other catalysts in Table 5. As a result of the study's goal of increasing yield, reducing reaction time, and using the bare minimum of catalysts. Also, ^1^HNMR data of products have been compared with literature (Table S1). (Table S1 has been added to the “Supporting Information” file).

**Table 5:** Analysis of the catalytic ability of the numerous catalysts in the text leads to the synthesis of **5a** and **5b**^a^**.**

Entry	Product	Catalyst	Conditions	Time/Yield	TON	TOF	References
1		I_2_	MeOH, rt	1 h/82%	8.2	0.13	^27^
2		Glycine	MeOH, rt	3 h/93%	9.3	0.05	^28^
3		Glutamic acid	MeOH, rt	2 h/91%	4.5	0.03	^34^
4		2,6-Pyridinedicarboxylic acid	MeOH, rt	1 h/85%	8.5	0.14	^37^
5		4CzIPN	blue LED (7 W), EtOH, rt	15 min/97%	97	6.46	This work
6		I_2_	MeOH, rt	1 h/81%	8.1	0.13	^27^
7		Glycine	MeOH, rt	3 h/90%	9	0.05	^28^
8		Glutamic acid	MeOH, rt	2 h/88%	4.4	0.03	^34^
9		2,6-Pyridinedicarboxylic acid	MeOH, rt	2 h/81%	8.1	0.06	^37^
10		4CzIPN	Blue LED (7 W), EtOH, rt	15 min/95%	95	6.33	This work

^a^Four-components are used in the synthesis: aniline, dimethyl/ethylacetylenedicarboxylate, and formaldehyde.

**Figure 5:**
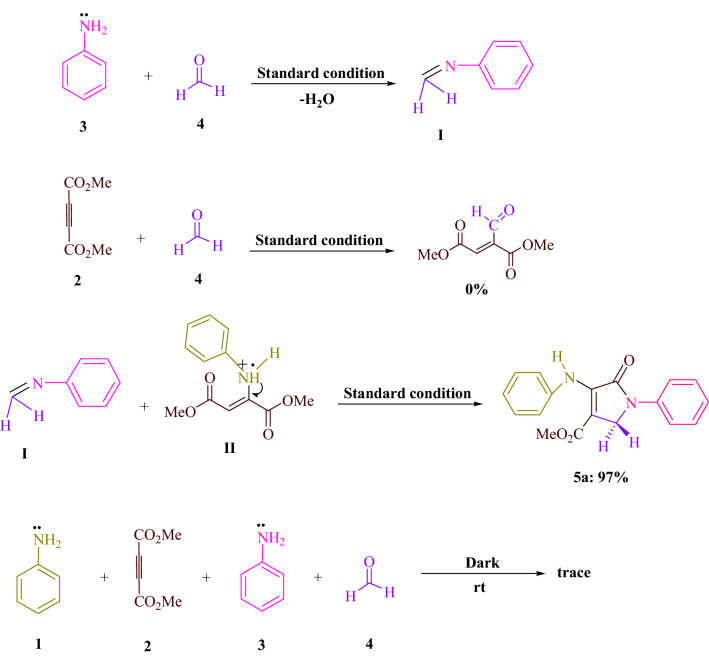
DMAD (**2**, 1 mmol), aniline (**1** and **3**, 2 mmol), and formaldehyde (**4**, 1.5 mmol) reactions provide important control studies for understanding their mechanism.

**Figure 6:**
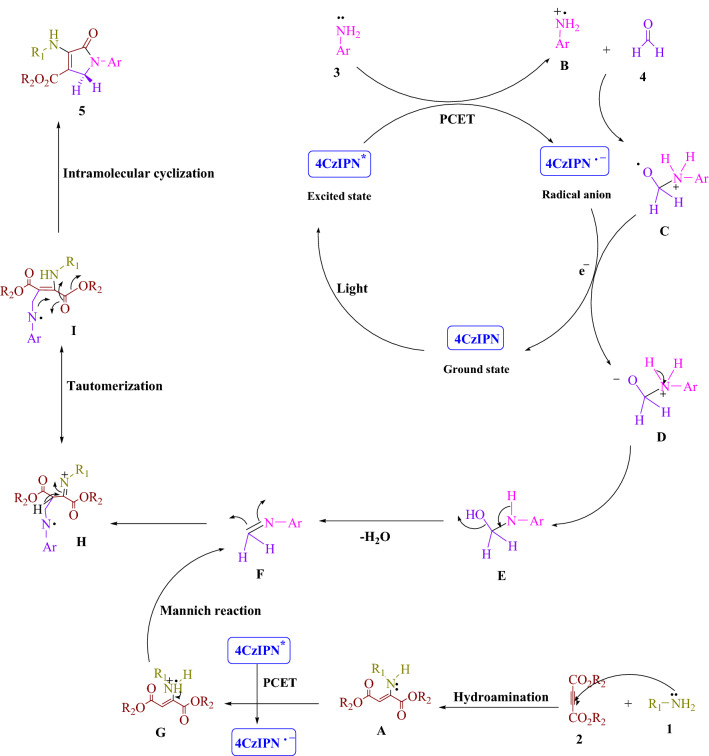
There was a detailed description of the mechanism of synthesis of polyfunctionalized dihydro-2-oxypyrroles.

In Fig. 5, Control experiments were carried out to reveal the mechanism behind this four-component reaction driven by visible light. Using standard conditions (4CzIPN in EtOH under blue LED), reducing H_2_O to obtain imine (**I**) was the procedure for condensation of aniline (**3**) with formaldehyde (**4**). Formaldehyde (**4**) and dimethyl acetylenedicarboxylate (DMAD) (**2**) did not react under identical reaction conditions. As a result, under normal conditions, 97% of the reactions between imine (**I**) and enamine radical (**II**) produced the predicted product **5a**. Even when the reaction was carried out in the dark, a trace of product **5a** was obtained. Based on the results of this experiment, Fig. 6 suggests a possible reaction route.

The proposed mechanism is illustrated in Fig. 6. 4CzIPN fluorophore organic dye created photocatalytic devices that use visible light as a renewable energy source by using the proton-coupled electron transfer (PCET) approach. Visible light accelerates the process. The Michael reaction produces enamine (**A**) by reacting amine (**1**) with dialkylacetylenedicarboxylate (**2**). By utilizing a PCET method and visible light irradiation, the aniline radical (**B**) is produced to enhance the visible-light-induced 4CzIPN^*^. In the next step, radical cation (**B**) reacts with formaldehyde (**4**) to form radical cation (**C**). By electron transfer (ET) process the radical adduct (**C**) and the 4CzIPN radical anion, intermediates (**D**), and the ground-state 4CzIPN are produced. The intermediate (**F**) is then obtained by removing one H_2_O molecule from (**E**). With a PCET approach, the enamine radical (**G**) is generated to enhance visible-light-induced 4CzIPN^*^. The Mannich reaction occurs between an activated imine (**F**) and an enamine radical (**G**), producing a more stable tautomeric form (**I**). Finally, a polyfunctionalized dihydro-2-oxypyrrole (**5**) is formed by intramolecular cyclization in intermediate (**I**).

In Table 5, several catalysts are compared in terms of their ability to catalyze polyfunctionalized dihydro-2-oxypyrroles. In view of its relatively minimal amount of photocatalyst, quick reaction time, and lack of byproducts, this technique can be used in visible light environments. At multigram scales, atom-economic protocols are very effective and influence the sector significantly.

## Conclusion

By using amines, dialkylacetylenedicarboxylases, and formaldehyde in radical Michael–Mannich reactions, polyfunctionalized dihydro-2-oxypyrroles were synthesized without the use of metals. Through proton-coupled electron transfer (PCET), the photosynthesis was catalyzed using a carbazole-based photocatalyst (4CzIPN), which is a novel donor–acceptor (D–A) fluorophore. The light from visible light can be used to generate renewable energy sources in an ethanol solution at room temperature and in an environment with air. As well as the fast reaction time and the lack of harmful solvents or catalysts, the process also takes advantage of small quantities of photocatalysts, outstanding yields, a high-efficiency reaction process, stable conditions, and renewable energy sources. It was not necessary to use chromatography for the separation process. A multigram scale reaction of model substrates can be accelerated without compromising the outcome. Therefore, the technique can be applied both commercially and environmentally.

## Supplementary Information

Supplementary Information.

## Data Availability

All data generated or analyzed during this study are included in this published article [and its supplementary information files].
