# Supplementary material for: Synthesis of polyfunctionalized dihydro-2-oxypyrroles catalyzed by 1,2,3,5-tetrakis(carbazol-9-yl)-4,6-dicyanobenzene (4CzIPN) as a novel donor-acceptor fluorophore
Source: Sci Rep. 2022 Oct 7;12:16911. doi: 10.1038/s41598-022-20689-4 (PMC9547064; doi:10.1038/s41598-022-20689-4)
Supplement: Supplementary file 1 — Supplementary Information. [file 41598_2022_20689_MOESM1_ESM.docx]

**Supporting Information**

**Synthesis of polyfunctionalized dihydro-2-oxypyrroles catalyzed by 1,2,3,5-tetrakis(carbazol-9-yl)-4,6-dicyanobenzene (4CzIPN) as a novel donor-acceptor fluorophore**

Farzaneh Mohamadpour^*^

School of Engineering, Apadana Institute of Higher Education, Shiraz, Iran

**Corresponding author.* [*mohamadpour.f.7@gmail.com*](mailto:mohamadpour.f.7@gmail.com)

**Table of content**

1. Spectra data
2. Spectra files
3. Tables
   1. Table S1. Comparison of ^1^HNMR data.
4. References
5. **Spectra data**

***Methyl4-(4-chlorophenylamino)-1-(4-chlorophenyl)-2,5-dihydro-5-oxo-1H-pyrrole-3-carboxylate (5e)***

Yield: 90%; m.p. 172-173 °C; ^1^HNMR (300 MHz, CDCl_3_): 3.74 (3H, s, OCH_3_), 4.50 (2H, s, CH_2_–N), 7.09 (2H, d, *J*= 8.4 Hz, ArH), 7.45 (2H, d, *J*= 8.4 Hz, ArH), 7.58 (2H, d, *J*= 8.4 Hz, ArH), 7.75 (2H, d, *J*= 8.4 Hz, ArH), 8.03 (1H, s, NH) ppm.

***Ethyl3-(4-chlorophenylamino)-1-(4-chlorophenyl)-2,5-dihydro-2-oxo-1H-pyrrole-4-carboxylate (5f)***

Yield: 87%; m.p. 165-167 °C; ^1^HNMR (300 MHz, CDCl_3_): 1.30 (3H, t, *J*= 9.6 Hz, OCH_2_CH_3_), 4.28 (2H, q, *J*= 9.6 Hz, OCH_2_CH_3_), 4.54 (2H, s, CH_2_–N), 7.10 (2H, d, *J*= 11.6 Hz, ArH), 7.31 (2H, d, *J*= 10.0 Hz, ArH), 7.39 (2H, d, *J*= 11.6 Hz, ArH), 7.77 (2H, d, *J*= 11.6 Hz, ArH), 8.07 (1H, s, NH) ppm.

***Methyl4-(4-bromophenylamino)-1-(4-bromophenyl)-2,5-dihydro-5-oxo-1H-pyrrole-3-carboxylate (5i)***

Yield: 84%; m.p. 180-182 °C; ^1^HNMR (300 MHz, CDCl_3_): 3.81 (3H, s, OCH_3_), 4.52 (2H, s, CH_2_–N), 7.04 (2H, d, *J*= 11.2 Hz, ArH), 7.46 (2H, d, *J*= 11.6 Hz, ArH), 7.53 (2H, d, *J*= 12.0 Hz, ArH), 7.71 (2H, d, *J*= 12.0 Hz, ArH), 8.06 (1H, s, NH) ppm.

***Ethyl3-(4-bromophenylamino)-1-(4-bromophenyl)-2,5-dihydro-2-oxo-1H-pyrrole-4-carboxylate (5j)***

Yield: 85%; m.p. 167-169 °C; ^1^HNMR (300 MHz, CDCl_3_): 1.29 (3H, t, *J*= 9.6 Hz, OCH_2_CH_3_), 4.28 (2H, q, *J*= 9.6 Hz, OCH_2_CH_3_), 4.53 (2H, s, CH_2_–N), 7.04 (2H, d, *J*= 11.6 Hz, ArH), 7.45 (2H, d, *J*= 11.2 Hz, ArH), 7.53 (2H, d, *J*= 12.0 Hz, ArH), 7.72 (2H, d, *J*= 11.6 Hz, ArH), 8.05 (1H, s, NH) ppm.

*Methyl4-(4-ethylphenylamino)-1-(4-ethylphenyl)-2,5-dihydro-5-oxo-1H-pyrrole-3-carboxylate (5m)*

Yield: 92%; m.p. 122-124 °C; ^1^HNMR (400 MHz, CDCl_3_): 1.26 (6H, t, *J*=2.4 Hz, 2CH_2_CH_3_), 2.67 (4H, q, *J*=7.2 Hz, 2CH_2_CH_3_), 3.76 (3H, s, 2OCH_3_), 4.53 (2H, s, CH_2_-N),7.09 (2H, d, *J*=8.4 Hz, ArH), 7.17 (2H, d, *J*=8.4 Hz, ArH), 7.24 (2H, d, *J*=8.8 Hz, ArH),7.70 (2H, d, *J*=8.8 Hz, ArH), 8.05 ( 1H, s, NH) ppm; ^13^CNMR (100 MHz, CDCl_3_): 15.6, 15.7 (2CH_2_-CH_3_), 28.3 and 28.4 (2CH_2_-CH_3_), 48.3 (CH_2_N), 51.3 (OCH_3_), 101.9, 119.4, 123.1, 127.8, 128.5, 136.1, 136.4, 140.8, 141.3, 143.6 (C_Ar_), 163.6 (C=O, amide), 165.1(C=O, ester); MS (EI) m/z (%): 364 (M , 59), 349 (1), 332 (10), 318 (4), 305 (100), 290 (1), 277 (7), 261 (4), 247 (2), 233 (2), 216 (13), 199 (2), 186 (1), 173 (14), 158 (12), 145 (3), 132 (18), 118 (10), 103 (12), 90 (8), 77 (21), 64 (2), 51 (4).

*Ethyl4-(4-ethylphenylamino)-1-(4-ethylphenyl)-2,5-dihydro-5-oxo-1H-pyrrole-3-carboxylate (5n)*

Yield: 94%; m.p. 101-103 °C; ^1^HNMR (400 MHz, CDCl_3_): 1.24 (9H, m, 3 CH_2_CH_3_), 2.67 (4H, q, *J*=7.2 Hz, 2CH_2_CH_3_), 4.22 (2H, q, *J*=7.2 Hz, CH_2_CH_3_), 4.54 (2H, s, CH_2_-N), 7.09 (2H, d, *J*=8.4 Hz, ArH), 7.16 (2H, d, *J*=8.4 Hz, ArH), 7.24 (2H, d, *J*=8.4 Hz, ArH), 7.71 (2H, d, *J*=8.8 Hz, ArH), 8.01 (1H, s, NH) ppm; ^13^CNMR (100 MHz, CDCl_3_): 14.2, 15.6 and 15.7 (3CH_2_-CH_3_), 28.3 and 28.4 (2CH_2_-CH_3_), 48.4 (CH_2_N), 60.3 (OCH_2_-CH_3_), 102.5, 119.5, 122.8, 127.8, 128.7, 136.3, 136.4, 140.7, 141.3 and 143.1 (C_Ar_), 163.6 (C=O, amide), 164.8 (C=O, ester); MS (EI) m/z (%): 378 (M , 2), 357 (2), 339 (3), 321 (4), 305 (5), 292 (7), 275 (4), 262 (7), 239 (12), 218 (5), 199 (11), 185 (5), 171 (8), 152 (10), 130 (25), 105 (35), 91 (93), 77 (57), 57 (56), 43 (100).

***Methyl 3-(benzylamino)-1-(4-bromophenyl)-2,5-dihydro-2-oxo-1H-pyrrole-4-carboxylate (5o)***

Yield: 88%; m.p. 118-120 °C; ^1^HNMR (300 MHz, CDCl_3_): 3.81 (3H, s, OCH_3_), 4.43 (2H, s, CH_2_–N), 5.13 (2H, d, *J*= 8.8 Hz, CH_2_–NH), 6.87 (1H, br s, NH), 7.29–7.38 (5H, m, ArH), 7.51–7.55 (2H, m, ArH), 7.68–7.73 (2H, m, ArH) ppm.

***Methyl 3-(benzylamino)-1-(4-fluorophenyl)-2,5-dihydro-2-oxo-1H-pyrrole-4-carboxylate (5u)***

Yield: 96%; m.p. 169-171 °C; ^1^HNMR (300 MHz, CDCl_3_): 3.81 (s, 3H, OCH_3_), 4.44 (s, 2H, CH_2_–N), 5.14 (d, 2H, *J*= 8.8 Hz, CH_2_–NH), 6.90 (br s, 1H, NH), 7.09–7.15 (m, 2H, ArH), 7.29–7.38 (m, 5H, ArH), 7.72–7.77 (m, 2H, ArH) ppm.

1. **Spectra files**


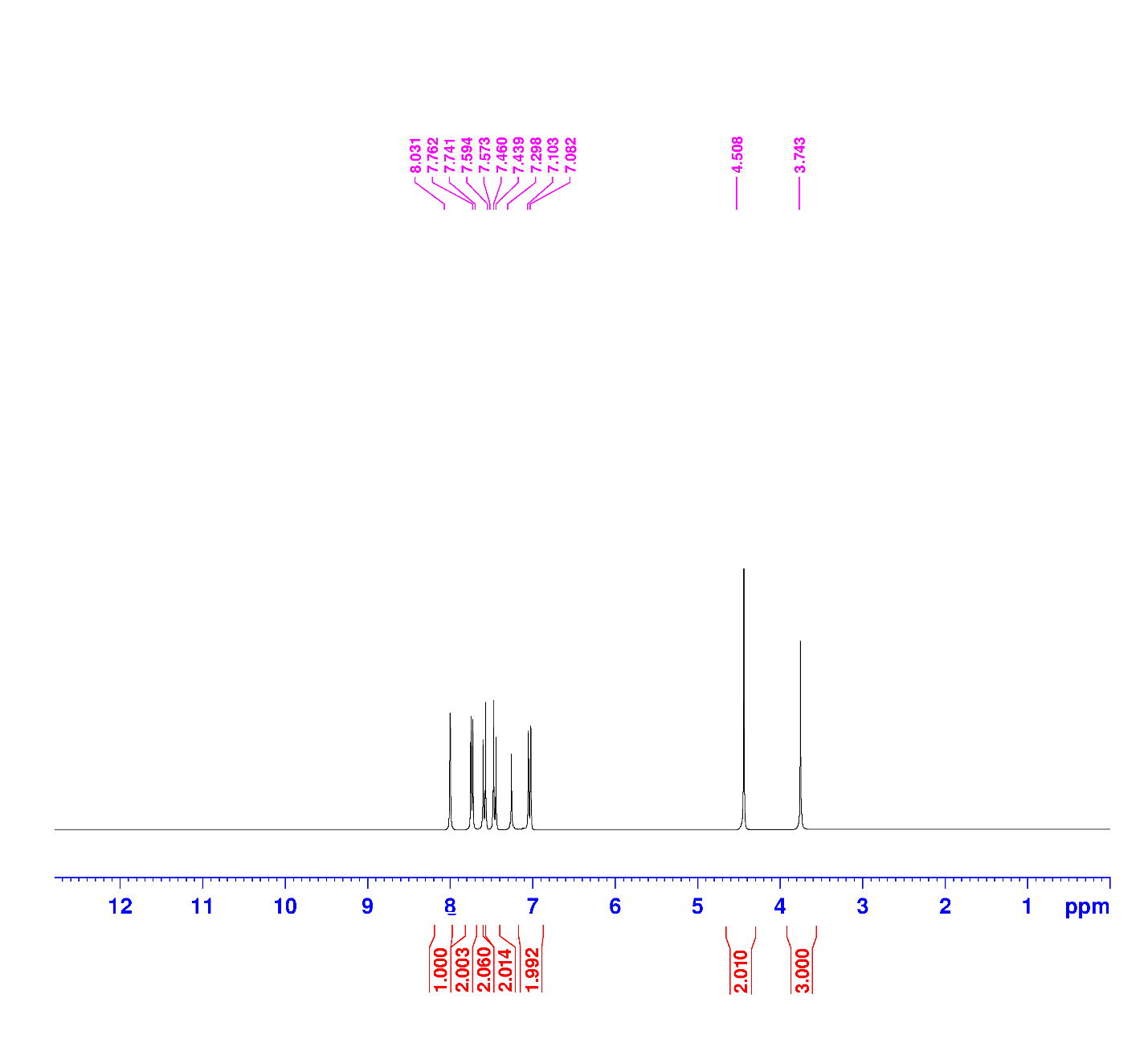


**Fig S1*.***^1^HNMR Spectrum of compound (300 MHz, CDCl_3_) of **5e**


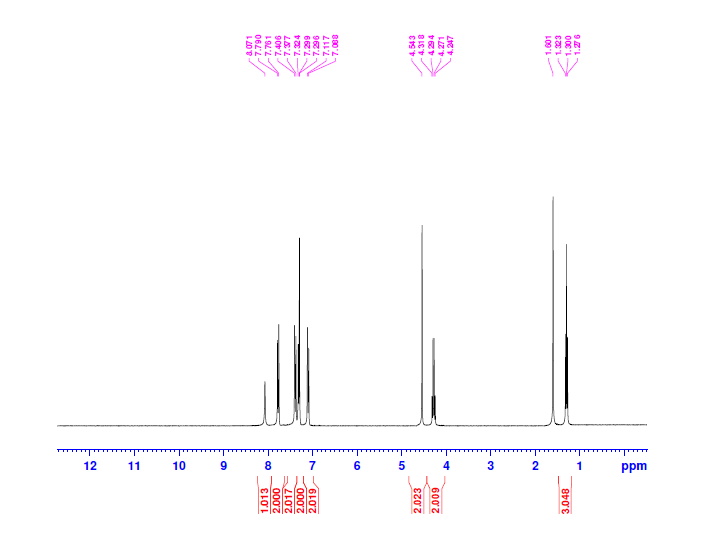


**Fig S2*.***^1^HNMR Spectrum of compound (300 MHz, CDCl_3_) of **5f**

**
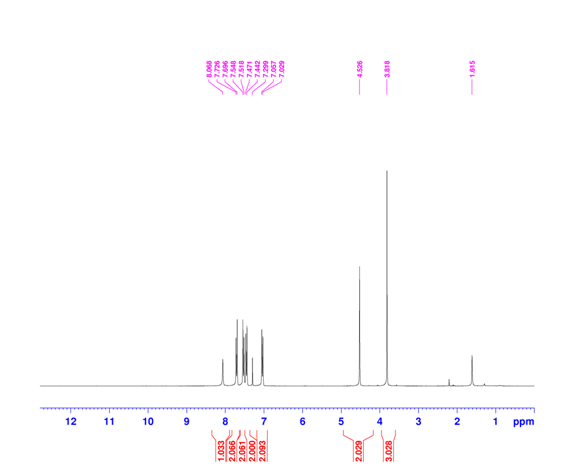
**

**Fig S3.** ^1^HNMR Spectrum of compound (300 MHz, CDCl_3_) of **5i**

**
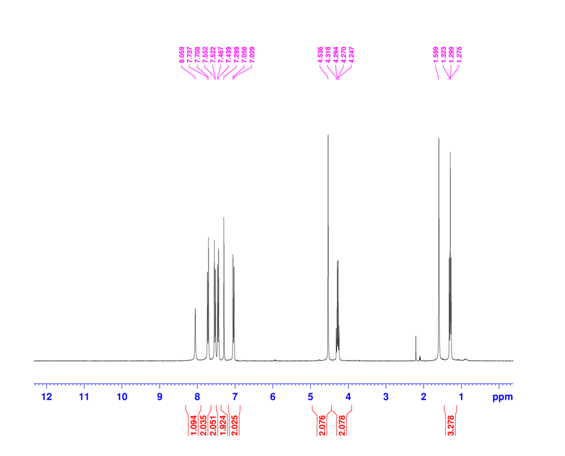
**

**Fig S4.** ^1^HNMR Spectrum of compound (300 MHz, CDCl_3_) of **5j**

**
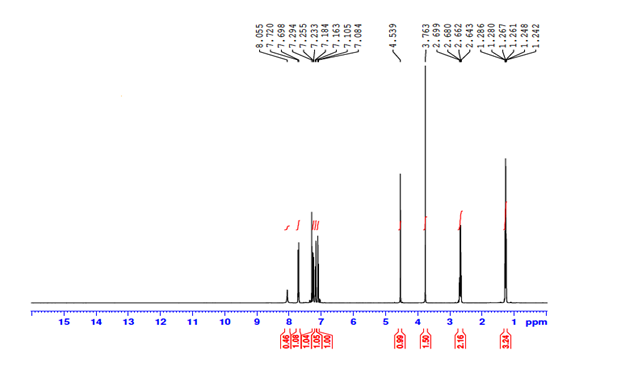
**

**Fig S5.** ^1^HNMR Spectrum of compound (400 MHz, CDCl_3_) of **5m**

**
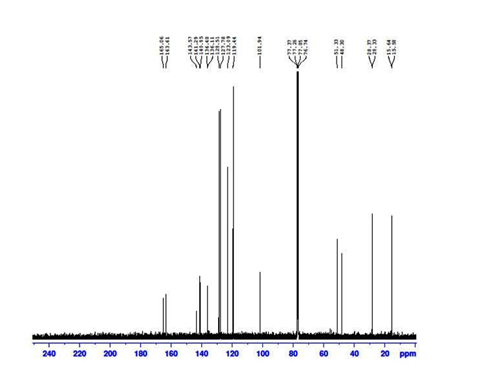
**

**Fig S6.** ^13^CNMR Spectrum of compound (100 MHz, CDCl_3_) of **5m**

**Fig S7.** Mass spectrum of compound **5m**

**
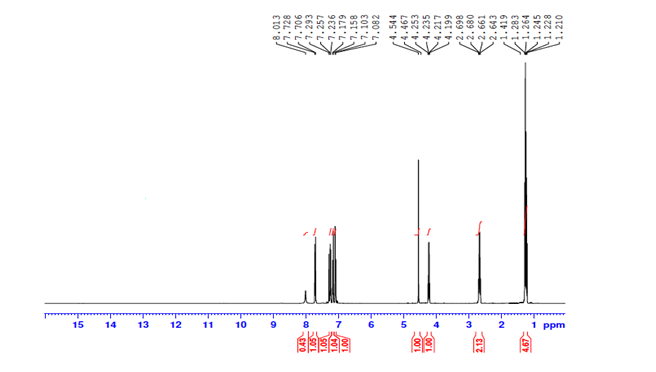
**

**Fig S8.** ^1^HNMR Spectrum of compound (400 MHz, CDCl_3_) of **5n**

**
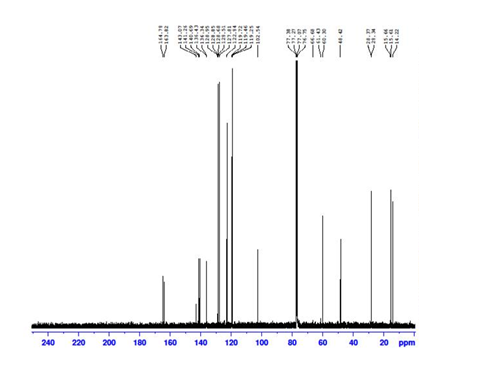
**

**Fig S9.** ^13^CNMR Spectrum of compound (100 MHz, CDCl_3_) of **5n**

**Fig S10.** Mass spectrum of compound **5n**

**
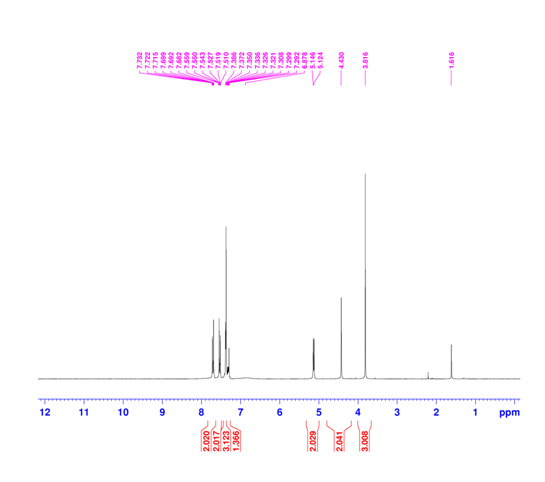
**

**Fig S11.** ^1^HNMR Spectrum of compound (300 MHz, CDCl_3_) of **5o**

**
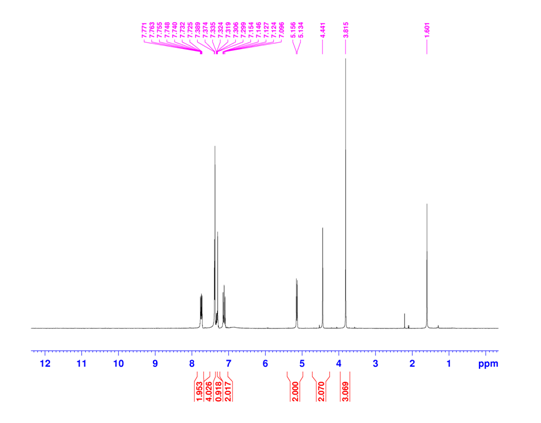
**

**Fig S12.** ^1^HNMR Spectrum of compound (300 MHz, CDCl_3_) of **5u**

1. **Tables**

| **Table S1.** Comparison of ^1^HNMR data for synthesis of polyfunctionalized dihydro-2-oxypyrroles. | | | | |
| --- | --- | --- | --- | --- |
| Entry | Product | H Shift (found) | H Shift (lit) | References |
| 1 |  | 3.81 (3H, s, OCH_3_)  4.52 (2H, s, CH_2_–N)  8.06 (1H, s, NH) | 3.79 (3H, s, OCH_3_)  4.48 (2H, s, CH_2_-N)  8.06 (1H, s, NH) | [1] |
| 2 |  | 1.29 (3H, t, *J*= 9.6 Hz, OCH_2_CH_3_)  4.28 (2H, q, *J*= 9.6 Hz, OCH_2_CH_3_)  4.53 (2H, s, CH_2_–N)  8.05 (1H, s, NH) | 1.29 (3H, t, *J* = 7.1 Hz, OCH_2_CH_3_)  4.27 (2H, q, *J* = 7.1 Hz, OCH_2_CH_3_)  4.52 (2H, s, CH_2_-N)  8.05 (1H, s, NH) | [1] |
| 3 |  | 3.81 (3H, s, OCH_3_)  4.43 (2H, s, CH_2_–N)  5.13 (2H, d, *J*= 8.8 Hz, CH_2_–NH)  6.87 (1H, br s, NH) | 3.77 (3H, s, OCH_3_)  4.41 (2H, s, CH_2_-N)  5.11 (2H, d, *J* = 6.4 Hz,  CH2-NH)  6.85 (1H, br, NH) | [2] |

1. **References**
2. L. Lv, S. Zheng, X. Cai, Z. Chen, Q. Zhu, S. Liu, Development of four-component synthesis of tetra-and pentasubstituted polyfunctional dihydropyrroles: free permutation and combination of aromatic and aliphatic amines, ACS Combinatorial Science 15 (2013): 183-192, <https://doi.org/10.1021/co300148c>.
3. N. Hazeri, S.S. Sajadikhah, M.T. Maghsoodlou, M. Norouzi, M. Moein, S. Mohamadian-Souri, Maltose, a natural, efficient and economical catalyst for the one-pot synthesis of highly substituted dihydropyrrol-2-ones, Journal of Chemical Research 37 (2013): 550-552.
